# Dataset of clinical, immunohistopathological and laboratory features of patients with MHC II deficiency suffering from enteropathy

**DOI:** 10.1016/j.dib.2019.104446

**Published:** 2019-08-28

**Authors:** Carsten Posovszky, Mehtap Sirin, Eva Jacobsen, Myriam Lorenz, Klaus Schwarz, Anjona Schmidt-Choudhury, Catharina Schütz, Manfred Hönig, Klaus-Michael Debatin, Ansgar Schulz, Peter Möller, Thomas F. Barth

**Affiliations:** a Department of Pediatrics and Adolescent Medicine, University Medical Center Ulm, Eythstr. 24, 89075, Ulm, Germany; b Institute for Transfusion Medicine, University of Ulm, 89081, Ulm, Germany; c Institute for Clinical Transfusion Medicine and Immunogenetics Ulm, German Red Cross Blood Service Baden-Württemberg – Hessen, 89081, Ulm, Germany; d Department of Pediatrics and Adolescent Medicine, Ruhr University Bochum, 44791, Bochum, Germany; e Department of Pathology, University of Ulm, 89075, Ulm, Germany

**Keywords:** MHC class II deficiency, Adaptive immunity, Mucosal immunity, Intestinal epithelial cells, Enteropathy, HLA-DR, Invariant chain, Hematopoietic stem cell transplantation

## Abstract

Major histocompatibility complex class II (MHC II) is essential for adaptive immune response. We recently reported on disturbed adaptive mucosal immunity due to MHC II deficiency and prolonged enteropathy. Here, we share medical history, flow cytometric analysis of blood lymphocytes, immunohistopathology, and fecal analysis of seven genetically confirmed patients with MHC II deficiency suffering from enteropathy. Data on flow cytometric analysis of HLA-DR expression on monocytes and B cells before hematopoietic stem cell transplantation (HSCT) and after *in-vitro* stimulation is shown. The course of immune reconstitution after HSCT of MHC II deficient patients in comparison to severe combined immunodeficiency (SCID) patients is described. In addition, immunohistopathology illustrating CD4 and CD8 T cell infiltration, absence of B lymphocytes and plasma cells, and disturbed immunoglobulin expression in the gut as well as absent HLA-DR expression in the liver is shown. Furthermore, data from fecal analysis such as stool fat, nitrogen, and water fraction as well as faecal markers such as alpha-1-antitrypsin, pancreas specific elastase 1, eosinophilic protein X (EPX), and beta defensin 2 are presented. Altogether this data demonstrates the complex phenotype of MHC II deficiency. The data can be valuable for researchers interested in mucosal immunity. For further interpretation of the data presented in this article, please see the research article “Persisting enteropathy and disturbed adaptive mucosal immunity due to MHC class II deficiency” (Posovszky et al., 2019).

**Table:** Specifications Table

Subject area	*Biology*
More specific subject area	*Clinical immunology and histopathology of MHC II deficiency*
Type of data	*image (microscopy), text file, graph*
How data was acquired	*Microscope, immunohistochemistry* *Flow cytometry* *Faecal analysis*
Data format	*Raw and analysed*
Experimental factors	*Tissue analysis, cell culture*
Experimental features	*Molecular and laboratory analysis, immunohistochemistry, flow cytometry*
Data source location	*Ulm, Germany*
Data accessibility	*data is with this article*
Related research article	*C. Posovszky, M. Sirin, E. Jacobsen, M. Lorenz, K. Schwarz, A. Schmidt-Choudhury, T. Rothoeft, C. Schuetz, M. Honig, K.M. Debatin, A. Schulz, P. Moller, T.F. Barth, Persisting enteropathy and disturbed adaptive mucosal immunity due to MHC class II deficiency, Clin Immunol, 203 (2019) 125–133.*https://doi.org/10.1016/j.clim.2019.04.012*. Epub 2019 Apr 24.**[1]*

**Table:** 

**Value of the data** • The data gives a comprehensive insight into the complex clinical, immunological and mucosal phenotype of MHC II deficiency. • This data extends our understanding of disturbed HLA-DR expression in different cell types or lymphocyte subsets in MCH II deficiency. • The data provide a basis for future exploration on the role of MHC II expression for adaptive immune responses in the context of gastrointestinal infections and oral tolerance induction. • This data will guide further investigations on the cross talk between epithelial cells and adaptive immune cells in the gut or lung mucosa.

## 1 Data

**Fig. 1:**
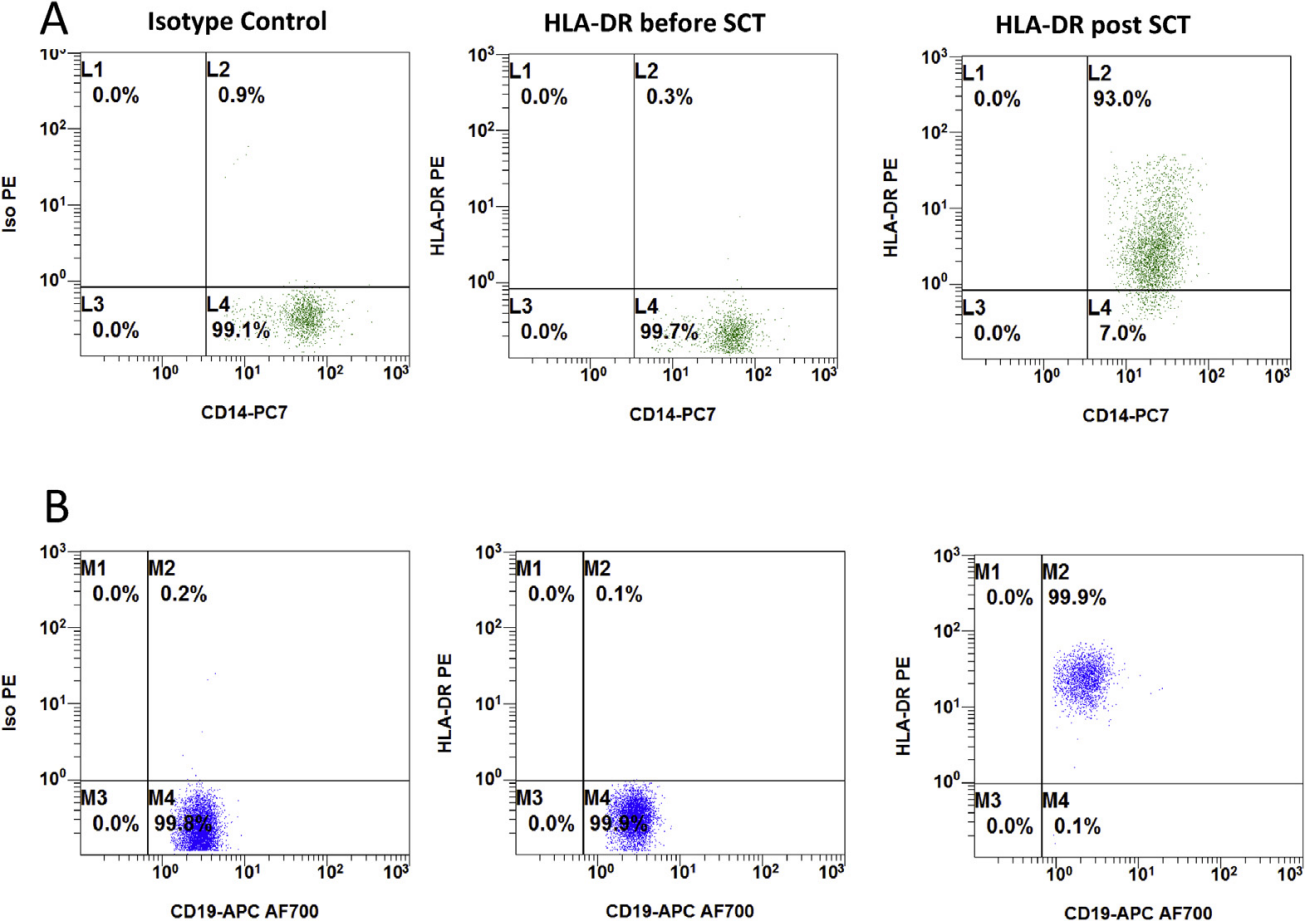
**HLA-DR expression on peripheral blood monocytes and B cells of patient 7 before and after HSCT:** Two-dimensional dot plot analysis of either PE-isotype control (left column) or HLA DR (middle and right column) expression plotted against the y-axis on unstimulated CD14^+^ monocytes (A) and CD19^+^ B cells (B) plotted against the x-axis. Expression pattern of patient 7 before HSCT (left and middle column) compared to HLA-DR expression after HSCT (right column) on CD14^+^ (A) monocytes and CD19^+^ B cells (B).

**Fig. 2:**
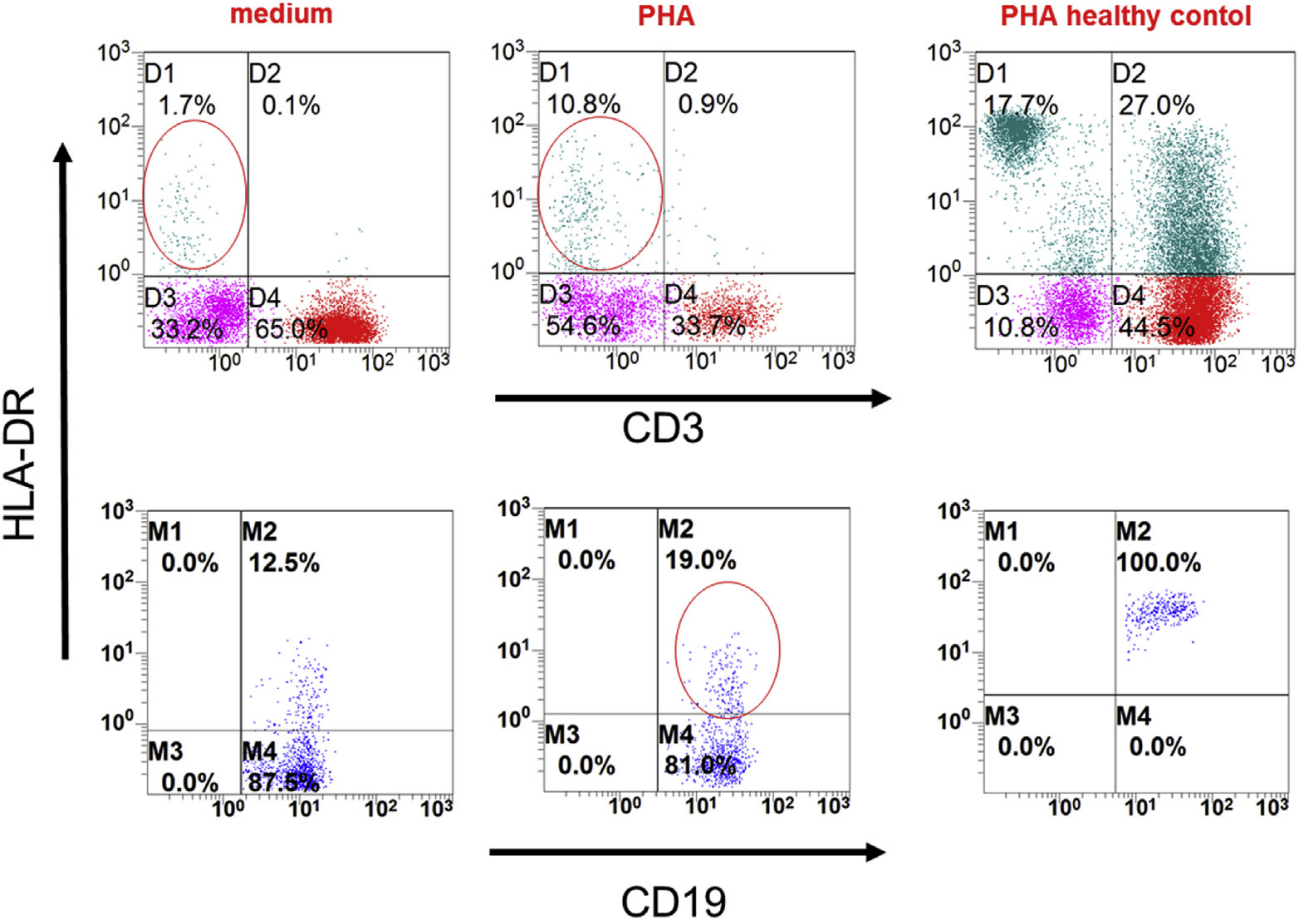
**Residual HLA-DR expression on stimulated peripheral B-cells of patient 1:** Peripheral blood mononuclear cells (MNCs) of patient 1 before HSCT were isolated by Ficoll density gradient. HLA-DR expression (HLA-DR PE) on CD3^+^ T cells (CD3 APC) (gated on lymphocytes, upper row) and CD19^+^ B cells (CD19APC AF700) (gated on CD19^+^ B cells, lower row) after 2 days of culture in RPMI medium containing 10% FCS at 37 °C (left column), medium enriched with 1.5% phytohaemagglutinin (PHA) (middle column). HLA-DR expression of healthy control MNCs after stimulation with PHA is shown in the right column. HLA-DR expression was restricted on CD3^−^lymphocytes (upper row red circles) and could mainly found on CD19^+^ B cells after stimulation (lower row red circle), albeit to a strongly reduced level in comparison with the healthy control. Percentages are indicated in the upper right quadrant of the dot blot analysis.

**Fig. 3:**
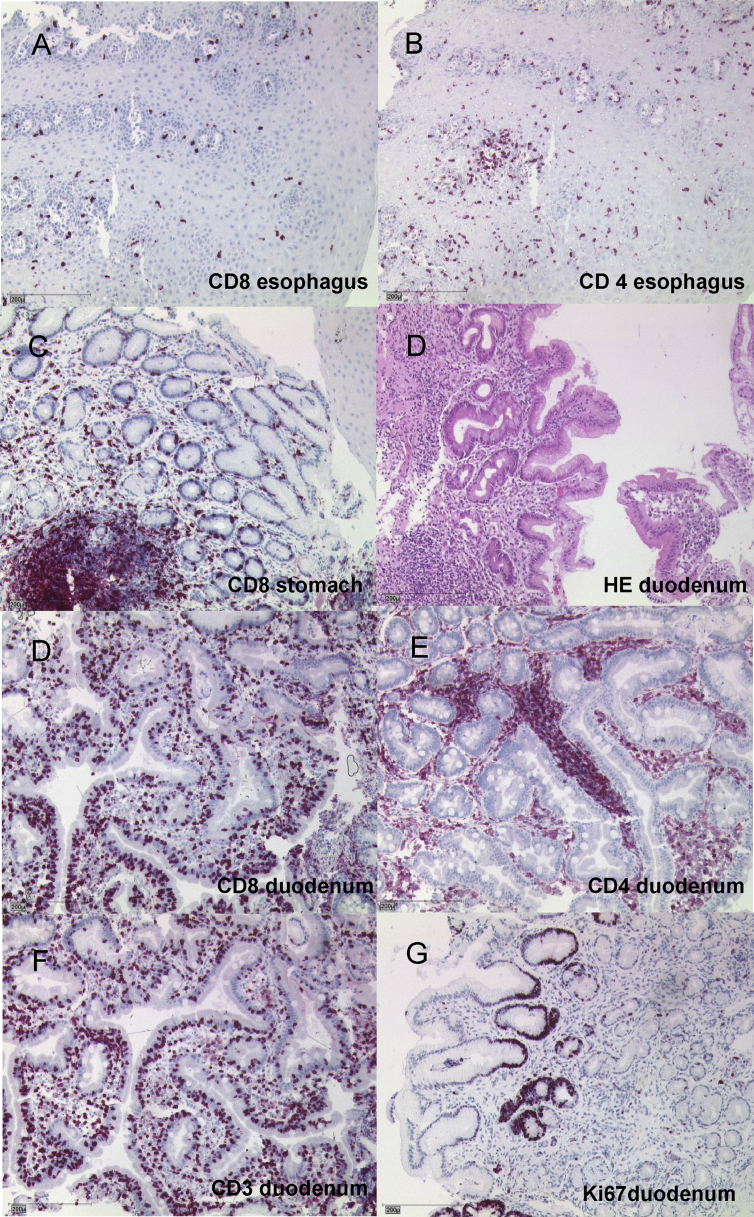
**Histopathology of patient 1 before HSCT:** Biopsies from the oesophagus (A, B), stomach (C) and duodenum (D–G) of patient 1 demonstrate extensive lymphocyte infiltrations and structural deformation. Pronounced villus destruction and crypt hyperplasia was seen in the duodenum (D–G). Intraepithelial lymphocytes (IEL) are increased up to 40% and are mainly CD8+(D), whereas CD4^+^ T cells are primarily found in the lamina propria and lymph follicle (E). Ki-67 staining shows a normal proliferation rate of the intestinal epithelial cells starting from the crypt base, with only few lymphoid cells being in the cell cycle (G). Black bars indicating 200 μm.

**Fig. 4:**
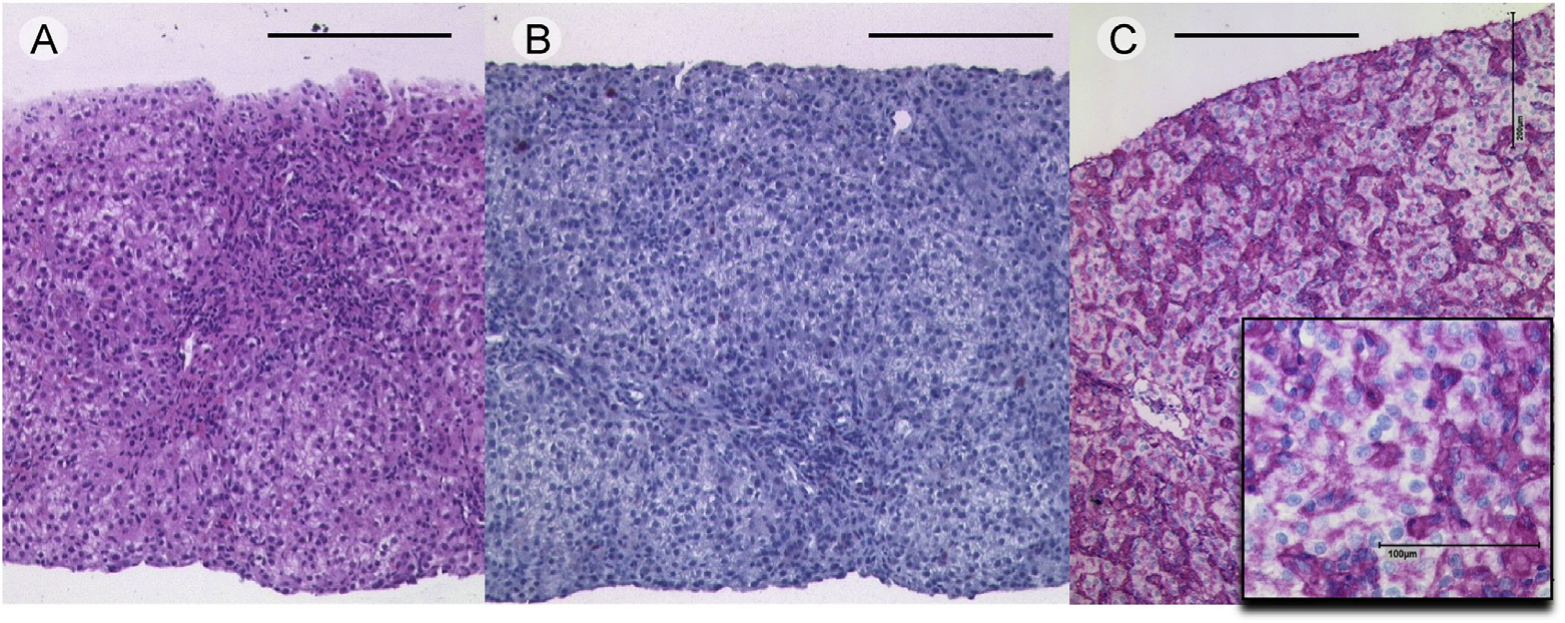
**Absent HLA-DR expression in the liver of MHC II deficient patient:** Liver biopsies of patient 4 demonstrate moderate chronic inflammation and moderate fibrosis without cholangitis as shown by HE staining (A). Immunohistochemically HLA-DR expressing lymphocytes are dramatically reduced (B). In addition, no HLA-DR was expressed in Kupfer cells, liver cells and cholangiocytes (B). In contrast, HLA-DR is strongly induced in sinusoidal lining cells in Epstein-Barr virus associated hepatitis (C). Black bars indicating 200 μm.

**Fig. 5:**
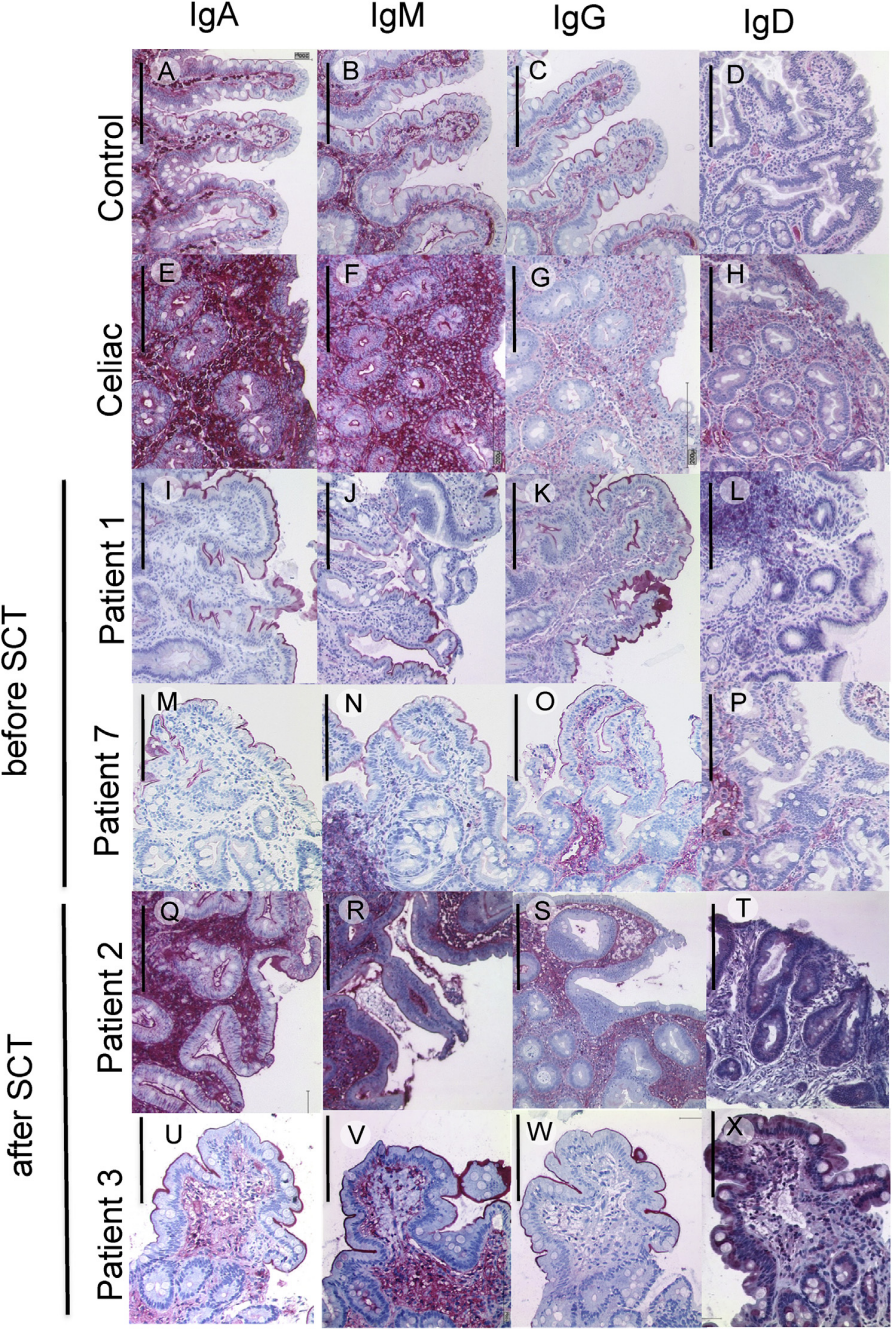
**Disturbed duodenal immunoglobulin expression in MHC II deficient patient:** Gluten-induced IgA, IgM, and IgD expression in a patient with celiac disease (E–H) versus a control patient with functional abdominal pain (A–D). In contrast, deficient IgA, IgM and IgG expression in MCHII deficient patient 1 (I–K) and 7 (M-O) is seen before HSCT, but detectable IgD in both patients (L, P). After HSCT IgA, IgM, IgG, and IgD is detectable in the lamina propria of patient 2 (Q, R, S, T) and IgA, IgM, and IgD in patient 3 (U, V, X). However, there is still reduced mucosal IgA and transportation across IECs of patient 3 (U). Polymeric IgA is produced by plasma cells in the lamina propria and binds to polymeric Ig receptor on the basolateral surface of IEC following endocytosis the receptor IgA complex passes the cellular components and is secreted into the lumen after proteolysis. Black bars indicating 200 μm.

**Fig. 6:**
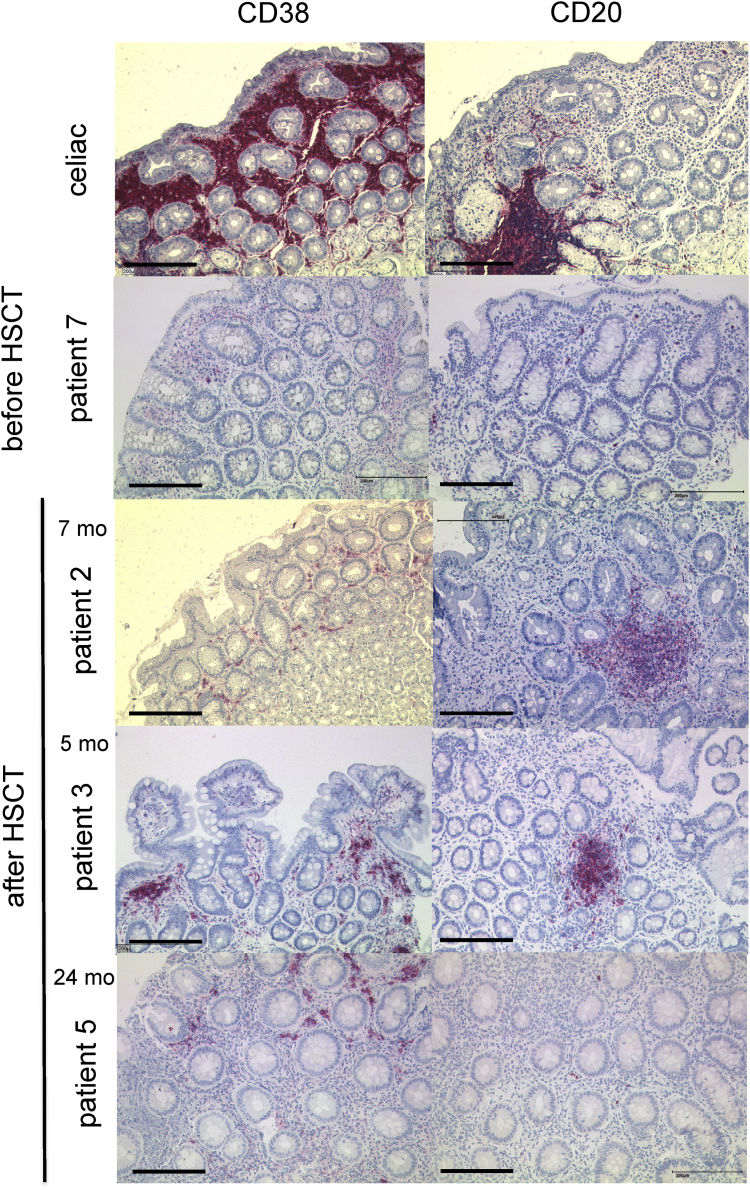
**Reduced CD20 and CD38 expressing cells in the lamina propria of MHC II deficient patient after HSCT:** CD20 and CD38 expression in a patient with celiac disease in the lamina propria and lymph follicle (first row). In contrast, rarely CD38 and CD20 expressing cells in patient 7 before HSCT (second row) and still reduced CD38 (left column) and CD20 (right column) expressing cells are seen in MCHII deficient patient 2, 3 and 5, still 5, 7 and 24 months after HSCT. Black bars indicating 200 μm.

**Fig. 7:**
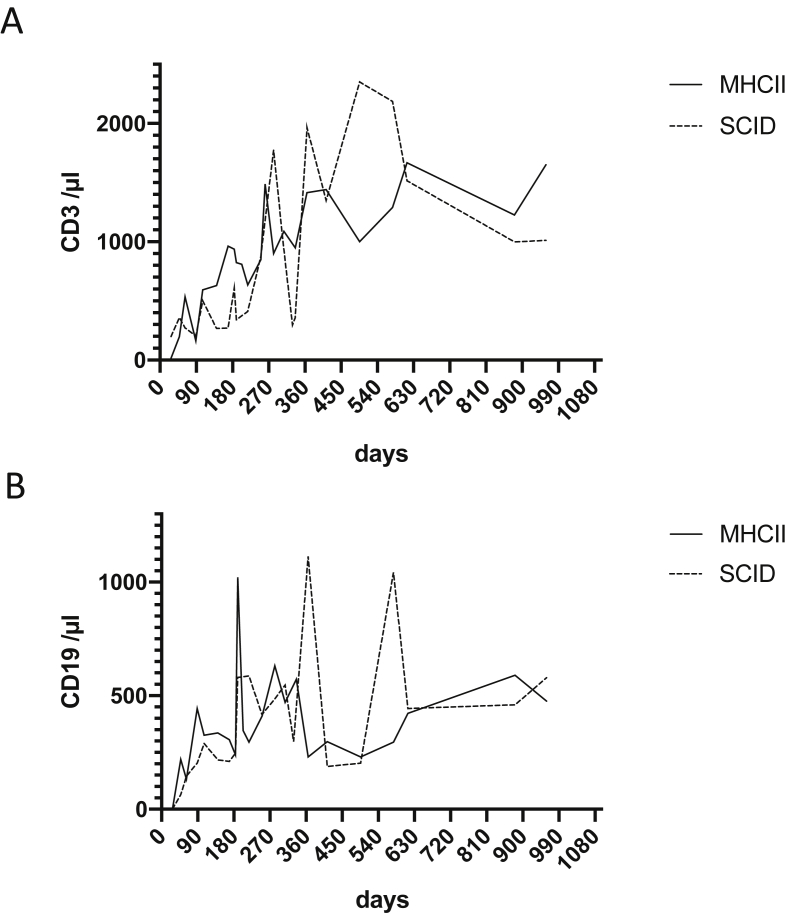
**Immune reconstitution of MHC II deficient compared with SCID patients after HSCT:** Time course (x-axis) of immune reconstitution after HSCT of absolute CD3^+^ (A) and CD19^+^ (B) peripheral blood lymphocyte counts of MHC II deficient patient 2, 3, 5, 6, and 7 (solid line) in comparison with five SCID patients (dashed line) are indicated. Grouped analysis of mean values of absolute counts per μl are presented.

**Fig. 8:**
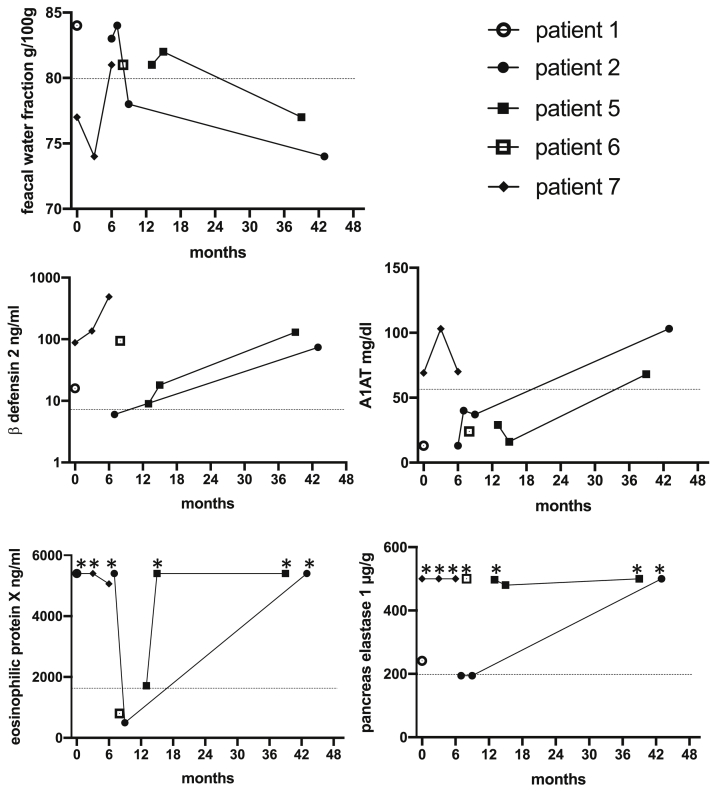
**Data of faecal analysis before and after HSCT:** Increased faecal water fraction indicate liquid stools and corresponds with diarrhoea. Interestingly, all patients displayed elevated β-defensin 2 levels in the faeces. This antimicrobial peptide is secreted from IEC as response to various bacteria into the gut. However, there is no or only mild loss of plasma alpha 1 antitrypsin (A1AT) into the faeces indicating a that there is no protein leakage. A1AT is elevated in patients with intestinal GvHD and decreasing levels correlate with treatment response. Eosinophilic protein X (EPX) is highly elevated in most patients before and after HSCT indicating inflammation and tissue damage (*maximum value > 5400 ng/ml). EPX is released from activated eosinophils and has cytotoxic characteristics. Specific pancreas elastase 1 is within the normal range (*maximum value > 500 μg/g) and indicates sufficient exocrine pancreatic function as well as digestive process. X axis displays months after HSCT and dotted line indicating cut-off values or lower limit for pancreas elastase-1 respectively.

This Data in Brief present a dataset of clinical, laboratory and immunohistochemical findings in patients with MHC II deficiency suffering from enteropathy. FACS analysis data show absent HLA-DR expression on peripheral blood monocytes and B cells of a MHC II deficient patient before hematopoietic stem cell transplantation (HSCT) and after reconstitution following HSCT (Fig. 1). In addition, flow cytometry data of residual HLA-DR expression on stimulated peripheral B cells of another patient with MHC II deficiency are presented (Fig. 2). This is complemented by immunohistology of lymphocyte infiltrations in the upper gastrointestinal tract of this patient before HSCT (Fig. 3). In addition, immunohistological staining demonstrating absent HLA-DR expression in the liver of another MHC II deficient patient with unclassified chronic liver disease is shown (Fig. 4). Immunobiological analysis illustrating disturbed duodenal immunoglobulin A, G, and M expression before and after HSCT are presented (Fig. 5). This is supplemented by immunohistology showing reduced CD20 and CD38 expressing cells in the lamina propria of MHC II deficient patient after HSCT (Fig. 6). Data of CD3 and CD19 counts of blood lymphocytes demonstrating a similar course of immune reconstitution after HSCT of MHC II deficient and SCID patients are shown (Fig. 7). Data of faecal analysis before and after HSCT show elevated β-defensin 2 and eosinophilic protein X (EPX), but only mild loss of alpha 1 antitrypsin and normal specific pancreas elastase 1 (Fig. 8).

## 2 Experimental design, materials and methods

### 2.1 Patients

We investigated seven patients out of six families born between 1998 and 2013 who were referred to Ulm University Children's Hospital. Written informed consent was obtained from the parents in accordance with the Declaration of Helsinki. The diagnosis of MHC II deficiency was confirmed by sequencing *RFXANK* and *RFXAP* genes.

### 2.2 Immunologic analysis of peripheral blood

Blood lymphocyte subsets were evaluated by flow cytometry (10-colour Navios, Beckman Coulter, Krefeld, Germany) using antibodies from Beckman-Coulter, Krefeld, Germany. MHC II expression on peripheral blood CD3^+^ T cells, CD14^+^ monocytes and CD19^+^ B cells was analysed (Navios v. 1.3). For analysis of stimulated lymphocytes, peripheral blood mononuclear cells were isolated by Ficoll density gradient and stimulated with 1,5% PHA-M (Gibco, California) for 2 days. Unstimulated control cells were cultured in media without PHA-M.

**Table:** Table of flow cytometry antibodies used:

Antibody	Fluorochrome	Clone	Dilution for cytometry	Provider
CD 3 IgG1 mouse	APC	UCHT1	1:20	Beckman Coulter, Krefeld, DE
CD14-IgG2a mouse	PC7	RM052	1:100	Beckman Coulter, Krefeld, DE
CD19-IgG1 mouse	APC-AF700	J3-119	1:20	Beckman Coulter, Krefeld, DE
CD45-IgG mouse	Krome-Orange	J33	1:20	Beckman Coulter, Krefeld, DE
HLA-DR IgG1 mouse	PE	Immu-357	0,2 μg/ml	Beckman Coulter, Krefeld, DE
Isotype IgG1 mouse	PE	DAK-G01	10 μg/ml	DAKO, Glostrup, DK

### 2.3 Mutation analysis

Peripheral blood mononuclear cells (PBMCs) of the patients were obtained after written informed consent of the parents. Genomic deoxyribonucleic acid (DNA) was extracted using standard methods. All exons, including the exon-intron boundaries of the *RFXANK, CIITA, RFX5, and RFXAP* genes (NM_000246, NM_003721, NM_00449, NM_000538) were amplified by polymerase chain reaction (PCR) analysis using specific primers.

Amplification and sequencing primers used were as follows:

**Table:** RFXANK:

Exon	Forward primer	Reverse primer
Exon 1-2 **	5′ CGCCGCACGGCTCCTGTTCC 3′	5′ GGTGCGATTGGTCCCAGTCCCA 3′
Exon 3 ***	5′ GCAGTCGGTGCCTACTTTAT 3′	5′ CAGGTTCCATGTGAGGCCTG 3′
Exon 4-5 *	5′ CAGATGGGCTTCTGTCCTGA 3′	5′ GTCATGAGTGGGTATGGACACCC 3′
Exon 6 *	5′ GGTGCAGCCTGGTGGTATTG 3′	5′ ACAAGGCGCCAGCAGACACAG 3′
Exon 7 ***	5′ GATGCGGCTGCTGTGGGTAC 3′	5′ ACTCTGTGAACATTTGAGGC 3′
Exon 8-9 ***	5′ GGTAAACCTTTGGTTTCTCCTGCC 3′	5′ GAACTCTTCAAGCAACACTTGG 3′
Exon 10 **	5′ GAGCGCAGGTTGGCCTGTGCCT 3′	5′ CCCTCATTCTGCACAGAAGG 3′

**Table:** CIITA:

Exon	Forward primer	Reverse primer
Exon 1 ****	5′ CATGTTGGCTTAGCTTGGCG 3′	5′ GCAGGACACAGACTCCTGTTCCC 3′
Exon 2–3 ****	5′ CCAGCTGGGAGTTGTTGTAGG 3′	5′ GACGTGGCTCATGATGAATGGG 3′
Exon 4-5 ****	5′ GGCCCAGAGGTTCCCCAGCCC 3′	5′ GCGAGGGAAGCAAGTTGGACCC 3′
Exon 6-7 ****	5′ GCCTGCTAGAGTCCTGAGCC 3′	5′ AGTTCCTGACAGTCCCTGCC 3′
Exon 8 ****	5′ AGAATCGCAAACACAGGTGC 3′	5′ GGGCACCACTGCCAAGTGC 3′
Exon 9 ***	5′ GTAGGGGTGACCCAAGTGCATT 3′	5′ GTGGATCTTATTTGAGACAATT 3′
Exon 10 ****	5′ CTAAGTGGCCCAGAGGGAGGGG 3′	5′ GCAATGTGAAGCCAGATATGGG 3′
Exon 11 ****	5′ GGTGGCAGTGCTGGCCTTGTGGTG 3′	5′ GTCAGGGCTCTGTCTTGGTG 3′
Exon 11 ****	5′ GAGCTGTCCGGCTTCTCCATG 3′	5′ AAAGAGACCCCACAGAGCACTG 3′
Exon 12 ***	5′ GGAGGACCCTTCATGGAGCTG 3′	5′ GGGCACTGGAAATGCCCATG
Exon 13 ****	5′ GCACCGCACCCAGACAGA 3′	5′ TGGGCAAGAGGGTAAGAATG 3′
Exon 14-15 ****	5′ GCCTTGTCTCTGCCCCACCCT 3′	5′ GAAAAAGTCAGCCGGACTTTG 3′
Exon 16 ****	5′ TGGCCACAGGACCTAACAGTATC 3′	5′ CCCCTGCAGCCAGCACGATGT 3′
Exon 17-18 ****	5′ GGAATCTAGGGATGGTGGCTTC 3′	5′ GAAGAGCATGATTTGAGCTC 3′
Exon 19 ****	5′ CTAGGCTGGGTGGAAGGAGG 3′	5′ CCATGCGTCCAAGGGTGGTGGCC 3′

**Table:** RFX5:

Exon	Forward primer	Reverse primer
Exon 1 ***	5′ GCCGGTACGAGCCCGGCGCC 3′	5′ GTGCACATGGGTGAAGAAGC 3′
Exon 2-4 **	5′ GGCATATGCAATGGTGAG 3′	5′ GAGATCAAGTGAGAGGCACA 3′
Exon 5-6 **	5′ GACACCCTGTAAGTCAGCTAG 3′	5′ GGCTTTATCAAGGCAGGAAG 3′
Exon 7-8 **	5′ GATCCCTAGGGACTCTTCG 3′	5′ TGTACATAATGACTGAGGCTG 3′
Exon 9-10 **	5′ GCTTTGGTCAATAGTCTTTG 3′	5′ AAAAGCAAGGGACCCAATTG 3′
Exon 11 **	5′ GACTTTGCTGTCCTGG 3′	5′ GGAGAATGGACTTCCTTAG 3′

**Table:** RFXAP:

Exon	Forward primer	Reverse primer
Exon 1 *****	5′ CGCCGCGCTCCCTGACAGCCC 3′	5′ TGGAAGTGGGGAGGCAGGCCC 3′
Exon 2 ***	5′ CCTGTTCATTACATCTTTGAAATACC 3′	5′ GATGTATAGTGTGATGTCATGAGC 3′
Exon 3 ***	5′ GCAGCTTAGTTGCAGTGCATC 3′	5′ CAAAGTGCAATACTTACTTATCCCC 3′

The PCR was performed with the following PCR conditions.

Initial denaturation step: 95 °C, 15 minutes.

Denaturation: 95 °C, 1 minute, 35 cycles.

Annealing: 1 minute, 35 cycles.

* Annealing temperature 54 °C.
** Annealing temperature 58 °C.
*** Annealing temperature 60 °C.
**** Annealing temperature 62 °C.
***** Annealing temperature 66 °C.

Elongation: 72 °C, 1 minute, 35 cycles.

Final elongation: 72 °C, 10 minutes.

The PCR products were directly sequenced in both directions using the ABI PRISM Terminator v1.1 Cycle Sequencing Kit (Applied Biosystems) and analysed on an ABI 3130xl Genetic Analyzer.

### 2.4 Histology and immunohistochemistry

Formalin fixed biopsy specimen were embedded in paraffin and sections of 3 μm were stained with hematoxylin-eosin (H&E), and for CD3 (F7.2.38, 1:100 dilution, DAKO, Glostrup, DK), CD4 (4B12, 1:200 dilution, DAKO), CD8 (C8/144B, 1:200 dilution, DAKO), CD20 (L26, 1:500 dilution, DAKO), CD38 (SPC32, 1:100 dilution, Menarini, Florence, IT), lgA (polyclonal, 1:4.000 dilution, DAKO), lgG (polyclonal, 1:30.000 dilution, Zytomed Systems, Berlin, DE), lgM (polyclonal, 1:10.000 dilution, DAKO), lgD (polyclonal, 1:1.000 dilution, DAKO), and HLA-DR (1B5, 1:2.000, Moldenhauer). Briefly, deparaffinized tissue sections were incubated with the respective antibody at a dilution of 1:50 using standardized protocols after pre-treatment for 10 minutes of the slides in a steamer. A commercially available detection system set was used (DAKO REAL detection systems, DAKO). For negative controls, the primary antibody was omitted.

**Table:** Table of immunohistochemical antibodies used:

antibody	clone	dilution for immunhistochemistry	provider	lot number
CD3	F7.2.38	1:100	Dako, Glostrup, DK	20033854
CD4	4B12	1:200	Dako, Glostrup, DK	20024494
CD8	C8/144B	1:200	Dako, Glostrup, DK	20015822
CD20	L26	1:500	Dako, Glostrup, DK	20023763
CD38	SPC32	1:100	Menarini, Florence, IT	6046949
IgA	polyclonal	1:4.000	Dako, Glostrup, DK	00065024
IgD	polyclonal	1:1.000	Dako, Glostrup, DK	00077870
IgG	polyclonal	1:30.000	Zytomed Systems, Berlin, DE	00086955
IgM	polyclonal	1:10.000	Dako, Glostrup, DK	00043013
HLA-DR	1B5	1:2.000	Gerd Moldenhauer, Heidelberg, German Cancer Research Center (DKFZ), DE	–

### 2.5 Quantitative analyses of faecal markers

The quantitative faecal analyses were carried out at the Institute of Microoecology, Herborn, Germany. Stool fat, nitrogen, and faecal water were measured by near-infrared reflectance analysis as described by Stein et al. [2]. Faecal β-defensin and eosinophilic protein X (EPX) were measured using the respective faecal tests of Immundiagnostik (Immundiagnostik AG, Bensheim, Germany). Faecal alpha-1-antitrypsin concentrations were measured using the AAT test (Maier Analytic, Sinsheim, Germany) following the providers' instructions. Concentrations of Pancreas specific elastase 1 was determined by the faecal elastase test-1 (ScheBo® Biotech AG, 35394 Giessen, Germany).

### 2.6 Clinical dataset of patient history

**Patient 1**, the 15-year old daughter of consanguineous parents presented with growth retardation below the first percentile adapted to age height percentile, delayed puberty, hepatosplenomegaly, and pneumonia. She developed juvenile-onset diabetes in the third year of life and thereafter hypothyroidism due to Hashimoto's thyroiditis with antibodies against thyroid peroxidase and thyroglobulin. In addition, a persistent massive lymphopenia and immunoglobulin deficiency was noticed making regular intravenous substitution of immunoglobulin necessary. She repeatedly developed gastrointestinal infections with various pathogens (see table 1 in Ref [1]). At 10 years an enteropathy with complete villous atrophy was diagnosed (MARSH IIIb) (see Fig. 2 in Ref [1]) and a gluten-free diet was initiated without success followed by an ineffective immunosuppressive therapy with azathioprine. Indeed, she was negative for antibodies against endomysium, gliadin, transglutaminase, autoimmune-enteropathy related antigen 75, adrenal gland, insulin, and islet cell. Other mutations leading to polyendocrinopathy and enteropathy such as IPEX and APECED were excluded. Sequencing revealed a novel homozygous c.761C>T mutation in exon 3 of the *RFXAP* gene leading to a premature stop codon (p.Gln251*). Therefore, the patient was diagnosed with MHCII deficiency. The patient died five months after HSCT due to a devastating adenovirus infection, campylobacter jejuni sepsis and multiorgan failure (see table 3 in Ref [1]).

**Patient 2** presented with massive diarrhoea from birth and failure to thrive from four months of life. After receiving all vaccinations until the age of 9 months she was found to excrete poliovirus. After severe bronchiolitis which led to mechanical ventilation at the age of 7 months the diagnosis of MHC II deficiency was made. She was dependent on long-term parenteral nutrition after HSCT due to intractable diarrhoea not responding to amino-acid based formula and not related to graft-versus-host-disease of the gut (GvHD). Her gastrointestinal infection with poliovirus resolved after 4 weeks following HSCT, whereas norovirus persisted for another 5 months (see table 3 in Ref [1]). In the following she also was suspected to have chronic pulmonary GvHD which was successfully treated with steroid pulses. More than 2 years after HSCT she had alopecia totalis which did not respond to local or systemic immunosuppression.

**Patient 3** started to have recurrent otitis from 2 ½ years of age on and was treated with antibiotics. He had a pneumonia due to pneumococcus infection and chronic rhinitis at 3 years of age. Thereafter he developed prolonged diarrhoea with 4 years of age and due to lymphopenia and lack of MHCII expression on lymphocytes the diagnosis of MHCII deficiency was molecularly confirmed by a homozygous RFXANK mutation. He received HLA identical HSCT from an unrelated donor with 5 years of age. Thereafter he suffered from a grade 3 GVHD of the skin and had long-term enteral feeding problems due to haemorrhagic type C gastritis and severe diarrhoea, which was not due to GvHD, but maybe related to gastrointestinal astro- and adenovirus infections and oral food intolerance (see table 3 in Ref [1]). He had a faecal loss of up to 3 L per day and received sandostatin for 4 days. In the course he still needed long-term parenteral nutrition. In addition, he had seizures and encephalopathy of unknown origin 6 weeks after HSCT, which was not due to infection. The last immunoglobulin substitution was on day 126 after HSCT.

**Patient 4** started to have recurrent sino-pulmonary infections, pneumonia and otitis media at the age of 9 months and received multiple courses of antibiotics. With 3 years of age the girl presented recurrent febrile illness, failure to thrive and massive chronic diarrhoea. The diagnosis of MHCII deficiency was based on a lack of HLA-DR expression on T cells and confirmed by a homozygous RFXANK mutation. She received regularly intravenous immunoglobulin substitution and antibiotic prophylaxis with Co-trimoxazole. Before her transplantation she developed hepatitis with liver fibrosis of unknown origin (Fig. 3). Three weeks after HLA compatible HSCT she died due to impaired coagulation, secondary thrombocytopenia, massive intracranial haemorrhage and arterial hypertension along with capillary leakage (see table 3 in Ref [1]).

**Patient 5** was given the diagnosis of MHCII deficiency already at three months due to a brother who died with 8 months of age of MHCII deficiency. The girl suffered from chronic diarrhoea, failure to thrive, hepatitis, and recurrent lower respiratory infections leading to early intravenous immunoglobulin substitution. After HSCT from an HLA-compatible unrelated donor she developed acute GvHD of the skin and of the gut. She needed multimodal immunosuppressive treatment for more than 3 years with frequent reactivations under tapering.

**Patient 6** suffered from a pulmonary infection with acute respiratory failure at the age of 4 months and 4 times yearly thereafter. She was diagnosed with MHCII deficiency at 7 years as her younger sister (patient 5) was presented for HSCT due to MHCII deficiency. In contrast to her sister, she did not display chronic diarrhoea and failure to thrive before HSCT (see table 1 Ref [1]), but she suffered from chronic diarrhoea and adenovirus infection thereafter (see table 3 Ref [1]). The post-transplant course was also complicated by severe haemorrhagic cystitis caused by Polyoma BK-virus.

**Patient 7** had recurrent pneumonia starting at the age of 8 months followed by chronic diarrhoea from 10 months (norovirus and Echovirus 13) onwards leading to failure to thrive and severe malnutrition with the necessity of parenteral nutrition (see table 1 Ref [1]). Reintroduction of liquids and solids was prolonged and a gastrostomy tube was placed for gradual progression in enteral nutrition (see table 3 Ref [1]). She received all regular vaccinations including BCG and measles until the age of 2 years without any side effects or complications.

## Acknowledgement

We thank Prof. Andreas Schwiertz from the Institute of Mikrooecology for his assistance in performing stool analysis.

## Appendix A Supplementary data

The following are the Supplementary data to this article:

Multimedia component 1 Multimedia component 1

Multimedia component 2 Multimedia component 2

## Conflict of interest

We confirm that there are no conflicts of interest associated with this publication and there has been no financial support for this work that could have influenced its outcome.
